# Antimalarial and Immunomodulatory Activities of *Tithonia diversifolia* (Asteraceae) Leave Flafonoids-Rich Extract Used in Cameroonian Traditional Medecine

**DOI:** 10.1155/2024/8645178

**Published:** 2024-06-26

**Authors:** Ntonifor Helen Ngum, Ndoah Ellen Masakebenagha, Oumar Mahamat

**Affiliations:** ^1^ Department of Zoology Faculty of Sciences University of Bamenda, North West, Bamenda, Cameroon; ^2^ Department of Microbiology and Parasitology Faculty of Sciences University of Bamenda, North West, Bamenda, Cameroon

## Abstract

**Background:**

Phytochemicals are considered the reliable source for the treatment of infection including malaria. Especially, phenols are known as potentially toxic to the growth and development of pathogens, among which flavonoids are the most extensively studied and play more intensive roles in ethnopharmacology. The immunological effect and role of *T. diversifolia* flavonoids-rich extract in treatment of malaria have therefore been examined in this study.

**Methods:**

*In vitro* test against *Plasmodium falciparum* and 4-day suppressive and Rane's tests against *Plasmodium berghei* in mice were used to evaluate the antimalarial activities. TNF-*α* and INF-*γ* levels, phagocytic tests, and production of oxygen and nitrogen radical were assessed to appreciate the immunomodulatory activity. One-way analysis of variance followed by post hoc Student's *t* tests was used for data analysis.

**Results:**

*T. diversifolia* flavonoids-rich extract at the concentrations ranging from 0.0004 mg/ml significantly (*p* < 0.05) inhibited in a concentration-dependent manner the growth of trophozoite up to 100% inhibition with 0.025 mg/ml at 24 and 48 hrs. Moreover, *T. diversifolia* flavonoids-rich extract reduced the level of parasitemia and improved in a dose-dependent manner the survival time of infected mice significantly (*p* < 0.05) compared to their control in 4-day suppressive test as well as in Rane's test. Additionally, *T. diversifolia* flavonoids-rich extract increased the TNF-*α* and INF-*γ* levels in rats infected by *P. berghei*. Furthermore, the flavonoid-rich extract enhanced weight of spleen in the rats, the metabolic and phagocytic activities of the peritoneal cells, and the concentration of nitric oxide and oxygen radicals in methylprednisolone-immunocompromised rats compared to the control (*p* < 0.05).

**Conclusion:**

The study has revealed that *T. diversifolia* flavonoids-rich extract through its antiplasmodial and phagocytic activities is a promising treatment of malaria.

## 1. Introduction

Malaria is one of the most devastating mosquito-borne diseases caused by several species of single-celled protozoan parasite known as *Plasmodium*. It remains an important disease affecting the world population. Consequently, one of the most important pillars of malaria control is the antimalarial therapy [1]. In sub-Saharan Africa, the proportion of patients utilizing antimalarial treatment outside the official circuit varies from 12 to 80% [2]. The extensive resistance to chloroquine and later to the antifolate drugs sulfadoxine-pyrimethamine led to the worldwide adoption of artemisinin- (ART-) based combination treatments (ACTs) as the first-line treatment of uncomplicated *P. falciparum* malaria in the early 2000s. However, the emergence of ART resistance is of significant concern.

Antimalarial drug efficacy is typically monitored by determining the in vivo therapeutic efficacy or in vitro/ex vivo sensitivity. However, in the developing world, people are exposed to the use of herbal decoctions for treatment of malaria. They are encouraged by the multiple actions of medicinal plants, directly through their antiplasmodial effect or by stimulating the immune response. Nitric oxide (NO) generated by phagocytes has antimicrobial activity demonstrated to kill *P. falciparum* in vitro [3]. Various antimalarial drugs are suspected to act via the production of reactive oxygen species (ROS) and nitric oxide (NO) production. Therefore, in addition to searching for antimalarial drugs, concerted research studies are also underway to boost the immune response against the parasite.

Over the past decades, the use of medicinal plant has become increasingly important in antimalarial therapy. *T. diversifolia* is one such plant. The leaves are traditionally used against malaria by boiling in water, and it is recommended as a candidate for antimalarial screening. In general, phytodrugs are one of the most important pillars of malaria control [4], and plants are of multiple chemical compounds, among which flavonoids, the polyphenolic compounds, are found to be highly antimalarial [5]. In previous studies, various bioactive compounds from *T. diversifolia* have been declared for significant therapeutic implications and favourable safety index [6]. However, from our knowledge, flavonoids of that plant were not investigated as antimalarial drugs. In order to identify the phytocompounds of this plant providing the same or better results as traditional healers in malaria treatment, the flavonoids-rich extract of leaf was assayed for its antimalarial and immunoregulatory activities. The results of this study would permit to validate the presence of these flavonoids with a potential antimalarial activity in *T. diversifolia* and the use of the plant as an effective therapeutic means in novel drug discovery.

## 2. Materials and Methods

### 2.1. Experimental Material

#### 2.1.1. Plant Collection and Identification

The fresh leaves of *T. diversifolia* were collected in the morning hours (9:30 am–10:30 am) from Nkwen, Bamenda III subdivision, in the Northwest Region of Cameroon. The plant was identified by botanists in the Department of Biological Sciences, University of Bamenda. The identification was authenticated by the National Herbarium at Yaounde, Cameroon. The sample was identified in comparison with the material of Dang Daniel no: 100 of National Herbarium No: 18591 SRF/Cam. The collected leaves were cleaned, sliced, and air-dried under shade at room temperature. The dried leaves were coarsely powdered. Then the powdered plant material was stored in a plastic container at room temperature until extraction.

#### 2.1.2. Extraction of Flavonoids

Flavonoid-rich extract of leaves from *T. diversifolia* was prepared as described by Krishnadhas et al. [5]. In summary, 10 g of the plant powdered was weighed and discarded into 100 mL of petroleum ether and incubated in an oven (40–60°C) for 24 hours; this was done to remove the lipids and sugars from the plants. The solid residue obtained was then treated with ethyl acetate for 24 hours and filtered. The resulting filtrate was concentrated using flash evaporator. The freeze-dried material was extracted with boiling acetone, and the residue was concentrated at atmospheric pressure. This concentrated residue was extracted successively with light petroleum ether (40–60°C) and benzene to remove nonflavonoid and other matter. The dry material, made of flavonoids (C6-C3-C6 compounds), was dissolved into a solvent solution with 1% Tween 80.

#### 2.1.3. Determination of Total Flavonoid Content

The total flavonoid content of the extract of leaves from *T. diversifolia* was quantified by following the procedure described by Krishnadhas et al. [5]. Into a test tube, 1 mL of the plant extract was put, and to it, 0.6 mL of sodium nitrite (5% w/v), 0.5 mL of aluminium chloride (10% w/v), and 3 mL of sodium hydroxide (4.3% w/v) were added and the mixture was added up with distilled water to make 10 mL. The reaction mixture was left to stand for 15 minutes, after which its absorbance was read at 500 nm in a semiautomated spectrophotometer. Quercetin was used as the standard, and the results were calculated as quercetin equivalence (quercetin eq., mg/ml) of the plant.

### 2.2. Collection of Blood Samples for In Vitro Sensitivity Test

A human blood sample was collected through venipuncture into an ethylenediaminetetraacetic acid (EDTA) tube and after washing three times by adding normal saline to the blood sample. After a centrifugation at 5000 RPM for 10 min, the supernatant was discarded and cells were washed three times with Roswell Park Memorial Institute Medium-1640 (RPMI-1640). Cells were suspended in RPMI-1640 to make a 20% erythrocyte suspension before the culture. RPMI-1640 was supplemented with l-glutamine (4.2 mM), piperazineethanesulfonic acid (HEPES) (25 mM), bovine foetal serum (10% (v/v)), and streptomycin (100 IU/mL) (complete RPMI-1640).

### 2.3. Harvesting of Murine Peritoneal Cells

Murine peritoneal cells were harvested from 4 male rats of 6 weeks old. Twenty-four hours before harvesting, rats were intraperitoneally injected with a volume of 1 ml of 25% starch solution to increase the number of cells in the peritoneal cavity. Rats were euthanised by cervical dislocation and the outer skin was cut to expose the inner skin in the peritoneal cavity. A volume of 5 ml of cold PBS containing 2.5% foetal bovine serum (FBS) was carefully injected into the peritoneal cavity (without puncturing any organs). Using a 10 ml syringe, cells were withdrawn and put into tubes and kept on ice. The collected cell suspension was washed by centrifugation (1800 tr/min, 10 min). The supernatant was discarded, and the cells were resuspended in RPMI. The viability of the cells (98.7%) was determined by mixing a volume of 100 *μ*l with an equal volume of 0.4% trypan blue staining dye and counted manually using a Malassez haemocytometer counting chamber under a light microscope.

### 2.4. Inocula

Three albino mice previously infected with *P. berghei* and having a parasitemia level of 20–30%, obtained from the Laboratory of Parasitology of the University of Dschang, were used as donors.

### 2.5. Mice and Inoculation

Albino Swiss mice (*Mus musculus*) of age range 4-5 weeks with 20 to 22 g bw were obtained from the animal house of the faculty of Science of the University of Yaounde I, Cameroon, to be used for antimalarial in vivo study. To infect the mice, blood was collected from the donor (*P. berghei*-infected mice) after cervical dislocation into heparinized capillary tubes having trisodium citrate (0.5%). Collected blood was diluted with normal saline (0.9%) following the parasitemia of the donor, and red blood cells (RBCs) from noninfected mice (normal mice) were added to obtain a proportion of 5 × 10^7^ infected erythrocytes/ml blood. Animals were then infected by an intraperitoneal (ip) injection of 0.2 mL of diluted blood. Animals were then randomly separated into five groups (negative control, positive control, and three test groups) comprising six animals each. Animals were kept under natural conditions (normal temperature and 12 hours cycle of light and darkness). They were fed standard mouse feed and sufficient water ad libitum.

### 2.6. Rats and Treatments

Considering the sign of reduction in *P. berghei* parasitemia and the increase of TNF-*α* and INF-*γ* by the extract of *T. diversifolia* flavonoids in mice, the question was explored whether the immunostimulatory activities are due to phagocytes by *T. diversifolia* flavonoids. To investigate on this issue, the phagocytic activities of *T. diversifolia* flavonoids were assessed in albino rats (Wistar strain) weighing 200–250 g and of age range 8–10 weeks obtained from the laboratory of physiology and pharmacology of the University of Dschang. They were divided into groups of five. Group I was made of normal rats (normal control) and no treatment was given. Group II was made of rats receiving a dose of methylprednisolone sodium succinate (MPSS) 40 mg/20 ml (1 ml/kg) and received distilled water (negative control). Group III contained MPSS-induced immunocompromised rats and received levamisole (2.5 mg/kg) and was taken as positive control. Groups IV and V (test groups) were made of MPSS-induced immunocompromised rats and were treated with 50 mg/kg and 100 mg/kg of the flavonoid extract, respectively. Extract and levamisole were administered via intragastric route by gavage for one week every 2 days. All animals have received on the first day of treatment an injection of Bacillus Calmette–Guérin (BCG). All animals were kept at room temperature (12 hours cycle of light and darkness) and fed standard feed and had free access to water (ad libitum).

### 2.7. Cytotoxicity Test Using Haemolysis Assay

The haemolytic effect of the flavonoid extracts of *T diversifolia* was evaluated in human erythrocytes as reported by Lópezet al. [7]. In brief, 5 mL of normal saline was dispensed into 3 test tubes. Thereafter, 5 mL of the extract was added to the first test tube and a 2-fold dilution was done. A tube containing 5 ml of 1% Tween 80 was taken as a baseline haemolytic control. Then, 5 mL of the washed RBCs (20% erythrocyte suspension) was dispensed into all the test tubes. The tubes were incubated at 37°C for 1 hr and the amount of haemoglobin produced was determined by reading the optical density using a spectrophotometer at 540 nm. The following formula was used to calculate the percentage of haemolysis:

(1) $$\begin{array}{c} \%\;Ha\text{emolysis}=\left(\frac{OD\text{ sample}}{\text{OD control}}\right)\times 100. \end{array}$$

### 2.8. Cytotoxicity Test on Murine Cells Using MTT Assay

Murine cells (1.5 × 106 cells/ml) were plated onto 96-well plates and incubated with the flavonoid extracts (6.3, 12.5, 25, and 50 *μ*g/ml) for 48 h at 37°C in incubator using the candle as sources of CO2. Cells pated in RPMI complete media only were taken as control, and cells cultured in presence of Tween 80 (15%) were used as positive control. After the 48 h incubation, 25 *μ*l of MTT solution was added to each well. The plate was incubated for 3 hours and 50 *μ*l of dimethyl sulfoxide was added to each well. The optical density (absorbance) was measured at 592 nm using a microplate.

### 2.9. In Vitro Cultivation of *P. falciparum* Isolates and Sensitivity Test

To study the sensitivity of *P. falciparum* against plant flavonoid extracts, an infected blood sample was freshly obtained from a *P. falciparum*-infected donor and was diluted with fresh uninfected human RBCs to have a parasitemia of 8000 *P. falciparum*/µl of blood. *P. falciparum* was cultured in 96-well plates as described by Das et al. [8]. The parasites were cultured in the presence of the flavonoids of *T. diversifolia* at different concentrations (0.05, 0.025, 0.0125, 0.0063, 0.0031, 0.0016, 0.0008, and 0.0004 mg/mL) in a final volume of 200 mL. In detail, plant flavonoid extract was dissolved in complete RPMI-1640 supplemented with 0.5% (v/v) DMSO and 0.1% (v/v) Tween 80. Through a two-fold serial dilution carried out across the plate, the extract at different concentrations was distributed in triplicate in 100 *µ*l. A reference drug, Combiate 80/480 mg/mL (artemether and lumefantrine 80/480 mg/ml tablet) dissolved in RPMI-1640, was used as positive control. Later, the suspension of parasitized RBCs (100 *µ*L) was added into the wells of the microplate. The plate was then incubated at 37°C in an incubator for 48 hrs. The parasitemia was determined using Giemsa stained thick smear on slides after 24 hrs and 48 hrs. To assess the effect of the extract, 20 *µ*L of the culture was collected from each well and used to prepare a thick smear. The films were stained with 10% Giemsa solution (pH, 7.3) and observed under the light microscope (100x). The number of trophozoites was counted in 5 fields. The percentage of inhibition or inhibitory effect was calculated as follows:

(2) $$\begin{array}{c} \%\text{ Inhibition}=100-S, \end{array}$$

where *S* = (No. of trophozoites in the test well/No. of trophozoites in control well) × 100.

### 2.10. Four-Day Suppressive Test against *P. berghei* in Mice

The test was carried out following the method reported previously [9]. The animals received intraperitoneally a standard inoculum of 1 × 10^7^*P. berghei*-infected erythrocytes on day zero (D0) and then divided into groups of five animals of 6 animals each. The doses of the extract were given to them orally for four consecutive days by gavage with the aid of feeding cannula. One group of mice was given 5 mg/kg/day chloroquine (positive control) and another group taken as control group received an equivalent volume of distilled water. The three remaining received the extract at different concentrations each. On the fifth day of the experiment, thin blood films were made from each mouse and the parasitemia level was estimated.

### 2.11. Curative Test against *P. berghei* in Mice

Rane's test [10] was used to evaluate the curative activity or schizontocidal activity of flavonoid-rich extract of *T. diversifolia* leaves against *P. berghei* in mice. Mice were inoculated, and after 72 hrs, they were divided into the different groups and treated with different doses of the extracts, chloroquine (5 mg/kg/day), and distilled water. Animals were treated for 5 days. Then, the parasitaemia level was monitored during the 5 days posttreatment, while the number of survival animal or the time of death in each group was monitored for 15 days of observation.

### 2.12. Parasitemia and Survival Percentage

In the *in vivo* test, the parasitemia was determined by counting the number of infected RBCs (a minimum of five fields per slide) using a light microscope. Percentage of parasitemia and percent of chemosuppression were calculated using the modified Peters and Robinson formula [11]:

(3) $$\begin{array}{c} \%\text{ Parasitemia}=\frac{\text{Numberof parasitizedRBC}}{\text{TotalnumberofRBCcount}}\times 100,\\ \%\text{ Suppression}=\frac{\%\text{ Parasitemia in Neg}.\text{ control}-\%\text{Parasitemia in study group}}{\%\text{ Parasitemia in Neg}.\text{ control}}\times 100. \end{array}$$

Lastly, the animals were followed for 15 days and their mean survival time (MST) was determined using the following formula [4]:

(4) $$\begin{array}{c} \text{MST}=\frac{\text{Total number of days mice survived }}{\text{Total number of mice}}. \end{array}$$

### 2.13. Plasma Level Determination of Interferon and Tumor Necrosis Factor in *P. berghei*-Infected Mice

To explore whether the reduction of the parasitemia is associated to the modulation of immune response by *T*. *diversifolia* flavonoids, plasma levels of INF-*γ* and TNF-*α* were measured in blood collected as indicators of proinflammatory response activation at day 4 after infection and treatment. Through heart puncture, blood was collected from all experimental mice using heparinised tubes and their plasma was collected after centrifugation (3000 rpm, 10 min). Sandwich ELISA commercial kit (Invitrogen®, Thermo Fisher Scientific, USA) was used to quantify the plasma levels of INF-*γ* and TNF-*α*. The assay was carried out following the manufacturer's instructions. The absorbance was measured by optical density at 450 nm in a Microplate Absorbance Reader. Using the standard curve, INF-*γ* and TNF-*α* levels were expressed as pg/mL.

### 2.14. Measurement of Phagocytic Index in MPSS-Immunocompromised Rats

Phagocytic index was assessed using the carbon clearance test as reported previously [12]. In this test, each rat was intravenously injected with diluted India ink at 100 *µ*L/10 g body weight. After 2 min (*T*_1_) and 10 mns (*T*_1_), respectively, blood specimens were collected from the rat retinal venous plexuses and then mixed with 2 mL of 0.1% sodium carbonate. The absorbance was measured at 600 nm on a double beam spectrophotometer with 0.1% sodium carbonate as the blank. The liver and the spleen were weighed on an electronic balance and its phagocytic index was calculated as follows:

(5) $$\begin{array}{c} K=\frac{\left(1\;g\text{ OD}1-1\;g\text{ OD}12\right)}{\left(T_{2}-T_{1}\right)}, \end{array}$$

where OD1 was for *T*_1_ and OD2 for *T*_2_.

Phagocytic index = ∛*k* × *A*/(*B*+*C*), where *A* is the body weight, *B* is the liver weight, and *C* is the spleen weight.

### 2.15. Assessment of Energy Metabolism and Oxygen Metabolite Production in MPSS-Immunocompromised Rats

After a week of treatment and 24 hrs after the last administration, rats were weighed and then sacrificed using diazepam and ketamine (ratio: 3 : 1). Peritoneal cells were harvested by washing the peritoneum in 3 mL of PBS and used for metabolism assay. Blood was collected by cardiac puncture and the serum was harvested to measure the production of nitric oxide and oxygen radicals. Spleen and thymus were collected and weighed using an electronic balance. The organ index was calculated using the following formula reported by Evans Ngwenah et al. [13].

(6) $$\begin{array}{c} \text{Relative weight}\left(\frac{\text{mg}}{g}\right)=\frac{\left(\text{weight of thymus or spleen}\right)}{\text{body weight}}. \end{array}$$

To evaluate the mitochondrial metabolism, a cell suspension (density: 1 × 10^3^ peritoneal cells/ml) was prepared and 2 ml of this suspension was mixed with 1 mL of MTT (3-(4,5-dimethylthiazol-2-yl)-2,5-diphenyltetrazolium bromide) solution (1% in PBS). After 2 hrs of incubation, 1N HCl (1 ml) was added and the optical density was read at 540 nm. The mean of the optical densities in treated animal was compared to that of control to evaluate the energy metabolism of the cells [13]. In addition, nitrite oxide production was measured using the Greiss reagent as reported by Mahamatet al. [12]. Serum sample (100 *µ*L) was mixed in equal volumes with the Griess reagent (mixture of sulfanilamide solution and N-1-naphthylethylenediamine dihydrochloride solution). The absorbance at 540 nm was then measured using a microplate spectrophotometer (VersaMax), and the concentrations of the nitrite were obtained using the standard curve obtained using NaNO_2_. The protocol reported by Pinakiet al. [14] was used to assess the level of reactive oxygen species. In brief, 100 *µ*l of plasma diluted 20 times in phosphate buffer was mixed with 1 mL of acetate buffer. Thereafter, 25 *µ*L of working chromogen solution (N, N-dimethyl-p-phenylenediamine sulphate, Aldrich, Sigma) was added and the absorbance was taken at 505 nm after 6 min using a spectrophotometer. The absorbance values obtained were compared to the curve obtained using H_2_O_2_.

### 2.16. Statistical Analysis

Experimental values were expressed as mean ± standard deviation of an experiment done in triplicate. Data were analysed using one-way analysis of variance, followed by post hoc Student's *t* tests. Levels of *p* < 0.05 were considered as indicative of significance. All calculations were carried out using the GraphPad Prism® V5.03 software (GraphPad Software Inc®, CA, USA).

## 3. Results and Discussion

### 3.1. Cytotoxicity Effect of *T. diversifolia* Flavonoids-Rich Extract against Red Blood Cells

The preliminary screening carried out on the cytotoxic effect of the flavonoid-rich extract of *T. diversifolia leaves* against red blood cells revealed a significant cytotoxic effect (Table 1). All the tested concentrations of the extract caused a red blood cell haemolysis in a concentration-dependent manner (*p* < 0.05). A percentage of 28.83% was observed with the highest tested concentration of 0.05 mg/mL.

### 3.2. In Vitro Cytotoxicity Assay Using Rat Peritoneal Cells

**Figure 1:**
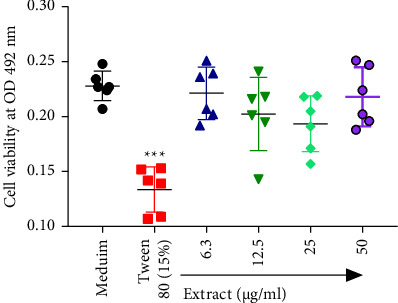
Effects of flavonoid-rich extract of leave from *T. diversifolia* on the viability of mouse peritoneal cells using the MTT assay. Results are expressed as mean ± SD and Turkey's multiple range test (*p* < 0.05) was used to compare the groups. ^*∗∗∗*^*p* < 0.001.

The flavonoids-enriched extract obtained from *T. diversifolia* leaves was tested for cytotoxic activity against immune cells obtained from rat's peritoneal cavity. The results of the MTT assay plate as presented in Figure 1 showed that *T. diversifolia* had no significant cytotoxic activity (*p* > 0.05) when compared to results for cells in medium only.

### 3.3. In Vitro Study of Susceptibility of P. falciparum to T. diversifolia Flavonoids-Rich Extract

The results of the flavonoid-rich extract of the *T. diversifolia* leaves on the *P. falciparum* during the in vitro test suggested that all the tested concentrations of the extract had a significant (*p* < 0.0001) inhibitory effect against the trophozoite stage after 24 hrs and 48 hrs (Table 2). The flavonoid-rich extract of *T. diversifolia* significantly decreased the parasite load in a concentration-dependent manner. Tested concentrations from 0.0031 mg/mL showed similar effect like the reference drug (artemether 80 mg/lumefantrine 480 mg) at 5.6 mg/ml (*p* > 0.05).

### 3.4. Suppressive Effect of *T. diversifolia* Flavonoids-Rich Extract

As shown in Table 3, the flavonoid-rich extract of *T. diversifolia* leaves showed a significant effect (*p* < 0.001) during the 4-day suppressive test. In comparison with the negative control group, the plant flavonoid extract significantly (*p* < 0.05) reduced the parasitemia in a dose-dependent manner. But still the standard drug (5 mg/kg/day) is more effective than the highest doses (100 mg/kg/day), which showed a chemosuppression of 66.40% fractions (*p* < 0.05).

### 3.5. Curative Effect of *T. diversifolia* Flavonoids-Rich Extract

Accordingly, the test doses of the flavonoid-rich extract of *T. diversifolia* leaves showed a significant effect (*p* < 0.001). In comparison with the negative control group, all the test doses of the extract reduce parasitemia significantly (*p* < 0.05). Besides, a dose-dependent parasitemia suppression effect was also observed. Similarly, the three extract doses prolonged the survival time of parasite-infected mice significantly (*p* < 0.001) in comparison with the negative control groups. However, the effect of the extract was lower than that of the group treated with artemether 80 mg/lumefantrine 480 mg (Table 4).

### 3.6. Effect of *T. diversifolia* Flavonoids-Rich Extract on Plasma Levels of INF-*γ* and TNF-*α* in Mice Infected with *P. berghei*

**Figure 2:**
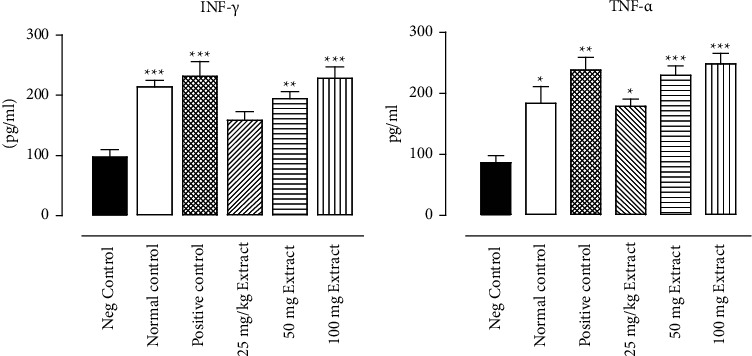
Plasma INF-γ and TNF-α levels in *P. berghei*-infected mice after 4 days of treatment with flavonoids-rich extract of *T. diversifolia*, artesunate or positive control (5 mg/kg), Neg. control, and normal animals. (a) INF-γ and (b) TNF-α. Values are expressed as pg/ml. The asterisks indicate significant difference compared to Neg. control.

The result indicated that the groups of animals who were given the *Tithonia diversifolia* flavonoids (50 and 100 mg/kg) and artesunate had a significantly high plasma level of INF-*γ* compared to infected animals not treated (Figure 2(a)). Similarly, in all groups of animals taking the *Tithonia diversifolia* flavonoids and artesunate, the TNF-*α* plasma levels were significantly higher than those in animals infected but not treated (Figure 2(b)).

### 3.7. Phagocytic Effect of Flavonoids-Rich Extract of T. diversifolia in MPSS-Immunocomprised Rats

The effect of the administration of flavonoids in MPSS-treated rats is presented in Table 5. Treatment with the extract significantly increased the size of the spleen as compared to the negative control. At 100 mg/kg, the effect of the flavonoid extract was similar to that of BCG vaccine. In contrast, the treatment with the flavonoid extract as well as the BCG did not affect the thymus index. Furthermore, treatment of the rats with flavonoid-rich extract increased the phagocytic index of the test animals compared to the animals that were treated with immunosuppressive drugs. The phagocytic index at 100 mg/kg was similar to that of animals treated with BCG vaccine. Treatment of rats with flavonoid-rich extract increased the cell metabolism of the animals compared to those kept without any treatment. At 50 mg/kg, the effect of the flavonoid extract was higher than that of BCG vaccine, and the results were significant at *p* ≤ 0.004. Also, flavonoid-rich extract of leaves of *T. diversifolia* significantly increased the serum NO of rats in a dose-dependent manner within one week of the treatment in rats compared to the serum level in control group. Similarly, injection of BCG increased serum level of NO in the rats. The total oxygen radical level in the serum of rats treated with the flavonoid-rich extract from *T. diversifolia* increased in a concentration-dependent manner. BCG was injected to the animals, and the level of oxygen radicals was increased compared to the serum level of oxygen radicals of the negative control (Table 5).

## 4. Discussion

Bioactive compounds found in the crude extract of the plant could produce their antimalarial effect via different mechanisms. Flavonoid compounds have been found to inhibit the growth and multiplication of the parasite that are essential for the control of the infection [15, 16]. Besides, phytochemicals like flavonoids and others may also exert their antimalarial effects indirectly by stimulating the immune system of the host [17]. Currently, there are no recommendations regarding the optimal content of flavonoids of *T diversifolia* and their subclasses in malaria treatment. However, many studies have evaluated the antimalarial activity of plant derivatives and indicated their efficacy against the *Plasmodium* species using in vivo and in vitro methods [18–20]. In the present study, the antimalarial effect of the flavonoid-rich extract of *Tithonia diversifolia* was evaluated by assessing its antiplasmodial and immunomodulatory activities. In the preliminary assay, the plant showed a toxic effect to the red blood cells as demonstrated by the hemolytic effect (28.83%) of the extract at 0.05 mg/ml. However, the extract did not show any toxic effect against murine peritoneal cells. This controversial effect may justify the reason why a low dose was recommended by the traditional healers. Despite the fact that the toxicity study reveals its safety, it is most important to perform a well-designed preclinical study to find a lead compound with desired efficacy for clinical study.

The potential antimalarial activities of the flavonoid-rich extract of *T. diversifolia* were evaluated in vitro against *P. falciparum* and in vivo using mice by evaluating the suppressive tests, standard models for the antimalarial screening [21]. Consequently, in the in vitro test, all the test doses of the flavonoid-rich extract of *T. diversifolia* inhibited the growth of trophozoite at 24 and 48 hrs in a dose-dependent manner, with the percentage of inhibition varying from 19 to 100%. Therefore, the plant flavonoid could have the potential of antiplasmodial activity [22, 23], specifically against the erythrocytic stages of the parasite. Besides, all the test doses of the flavonoid-rich extract of *T. diversifolia* inhibited the level of parasitemia and improved the survival time of infected mice in a dose-dependent manner in 4-day suppressive test as well as in Rane's test. Therefore, the flavonoid extract of *T. diversifolia* could have schizontocidal activity and could be used in preventive and curative treatment of malaria infection. The results of this study are in agreement with those of Afolayan et al. [24] who demonstrated the antimalarial effect of *T. diversifolia* in combination with other plants. The results showed that the percentage suppression was relatively lower during established infection (Rane test) than its effect in the 4-day suppressive test, and the flavonoid extract of leaves of *T. diversifolia* still showed a promising curative potential in established infection, justifying the probable rapid action of the extract during Rane's test. Taken together, the results of Rane's test suggest that the flavonoid-rich extract of leaves of *T. diversifolia* has therapeutic potential against established malaria infection since it is desirable to have both suppressive and curative activities in a phytodrug [21]. In both models (4-day suppressive test and Rane's test), the percentage suppression of parasitemia is ≥30% and such compounds are considered active [25].

In this study, TNF and INF levels, measured on day 4 after infection, showed high levels in infected mice receiving the extract. This may indicate that *T. diversifolia* flavonoid-rich extract stimulates specific patterns of innate immune response to infection with *P. berghei*. In various mouse strains, studies carry out with *P. yoelii* showed that IFN-*γ* and TNF produced 24 h after infection by *γδ* T lymphocytes and natural killer cells are indispensable for macrophage activation and early control of parasitaemia [26]. This may justify the reduction of the parasitemia in mice.

Studies demonstrated that when infected blood was inoculated in healthy mice, elimination of *Plasmodium-*infected erythrocytes occurs mainly in the spleen by monocyte-macrophage [27]. In this study, the administration of *T. diversifolia* flavonoid-rich extract along with the injection of BCG resulted in enhanced weight of spleen in immunocompromised rats. This demonstrates that the flavonoid extract of *T. diversifolia* can be helpful to boost the immune response. The plant flavonoid could have the potential of immunostimulatory activity. Spleen and thymus indices are considered to be the most elementary and conventional indices, which have been generally greatly exploited to evaluate the whole immune state of the organism [28], and compounds can be considered immunostimulatory active when there is increase in the indices of such organs [29, 30]. Consequently, the flavonoid-rich extract of leaves of *T. diversifolia* was presumed to be active. Besides, it was observed that peritoneal cells (neutrophils, monocytes, and macrophages) of mice treated with the flavonoid-rich extract of *T. diversifolia* have high metabolic and phagocytic activities. As phagocytes are the main participants in the innate immune response among the earliest cell types to respond to invasion by pathogenic organisms [31, 32], it may be intriguing that flavonoid-rich extract of leaves of *T. diversifolia* can be helpful to stimulate the immune response and consequently control the infectious diseases such as malaria infection [12]. Additionally, the results of the study showed that the serum has high concentration of NO and oxygen radicals in methylprednisolone-immunocompromised rats treated with the extract. NO and oxygen radicals are part of substances produced during intracellular killing mechanism in phagocytes. Therefore, these results are additional evidence demonstrating that the flavonoid-rich extract of *T. diversifolia* could have immunostimulatory activity and could be used in preventive and curative treatment of malaria infection.

## 5. Conclusion

According to this study, it can be concluded that that the flavonoid-rich extract of *T. diversifolia* is a good candidate to use in treatment of malaria as demonstrated here by its schizontocidal, antimalarial, and immunostimulatory activities. In this study, the crude flavonoids-rich extract was used. This may not provide the real efficacy of the extract. More investigations must be performed to assess the safety and use of *T. diversifolia* flavonoids and to identify the concerned types of flavonoids.

## Figures and Tables

**Table 1:** Haemolytic percentage of flavonoid-rich extract of *T. diversifolia* leaves against human red blood cells.

Samples	% haemolysis	Optical density
Normal saline	0 ± 0.00^a^	0.15 ± 0.02
Tween 80 (1%)	100 ± 0.00^b^	4.89 ± 1.05
0.05 mg/ml extract	28.83 ± 0.22^c^	1.36 ± 0.15
0.025 mg/ml extract	22.35 ± 1.51^d^	1.06 ± 0.95
0.0125 mg/ml extract	10.69 ± 3.64^e^	0.50 ± 0.05

Data are expressed as mean ± SD (test done in triplicate). Superscript letters indicate the difference within the group following Student's post hoc test with *p* < 0.05.

**Table 2:** Inhibition percentage of flavonoid-rich extract of *T. diversifolia* leaves against *P. falciparum* at 24 hrs and 48 hrs of incubation.

Treatment	% inhibition	
	24 hrs	48 hrs
Medium	0	0
Positive control (5.6 mg/ml)	91.86 ± 0.94^a^	95.45 ± 0.92^a^
Extract (mg/ml)		
0.05	100 ± 0.0^a^	100 ± 0.0^a^
0.025	100 ± 0.0^a^	100 ± 0.0^a^
0.0125	94.76 ± 2.76^a^	93.75 ± 1.46^ab^
0.0063	82.84 ± 9.22^ab^	88.63 ± 3.59^ab^
0.0031	79.36 ± 7.37^ab^	82.38 ± 8.42^ab^
0.0016	68.02 ± 12.82^b^	68.46 ± 12.46^b^
0.0008	21.51 ± 4.69^c^	20.45 ± 7.92^c^
0.0004	19.47 ± 6.46^c^	26.98 ± 6.31^c^
*p* value	*p* < 0.0001; *F* = 142.36	*p* < 0.0001; *F* = 163.13

Data are expressed as mean ± SD (test done in quadruplicate). Superscript letters indicate the difference within the group following Student's post hoc test with *p* < 0.05. Positive control: artemether 80 mg/lumefantrine 480 mg (5.6 mg/ml).

**Table 3:** Parasitemia and survival time of infected mice treated with the flavonoid-rich extract of *T. diversifolia* leaves in the 4-day suppressive test.

Groups	% parasitemia	% chemosuppression
Neg. control	*5*9.24 ± 8.30^a^	—
Positive control (5 mg/kg)	0.75 ± 1.23^b^	98.74
25 mg/kg extract	33.79 ± 6.70^c^	42.96
50 mg/kg extract	27.03 ± 4.62^cd^	54.36
100 mg/kg extract	19.90 ± 5.08^d^	66.40
	*F* = 83.88; *p* < 0.0001	

Data are expressed as mean ± SD (*n* = 6). Superscript letters indicate the difference within the group following Student's post hoc test with *p* < 0.05. Positive control: infected mice receiving artemether 80 mg/lumefantrine 480 mg (5 mg/kg); Neg. control: infected mice receiving distilled water.

**Table 4:** Parasitemia and survival time of infected mice treated with flavonoid-rich extract of *T. diversifolia* leaves in the 4-day suppressive test.

Groups	% parasitemia	% chemosuppression	Survival time (days)
Neg. control	67.64 ± 12.51^a^	—	8.17 ± 2.64^a^
A-L (5 mg/kg)	21.75 ± 5.74^b^	67.84	25.17 ± 4.40^b^
25 mg/kg extract	55.78 ± 13.62^ac^	17.53	15.00 ± 5.10^ac^
50 mg/kg extract	38.75 ± 8.46^bc^	42.71	21.00 ± 7.24^bc^
100 mg/kg extract	26.18 ± 10.77^b^	61.29	24.83 ± 4.92^b^
	*F* = 19.04: *p* < 0.0001		*F* = 12.18; *p* < 0.0001

Data are expressed as mean ± SD (*n* = 6). Superscript letters indicate the difference within the group following Student's post hoc test with *p* < 0.05. Positive control: infected mice receiving artemether 80 mg/lumefantrine 480 mg (5 mg/kg); Neg. control: infected mice receiving distilled water.

**Table 5:** Spleen and Thymus Index, Phagocytic Index and Serum Level of Total Oxygen Radicals and Nitric Oxide (NO) in Rats Treated with the Flavonoid-Rich Extract of *T. diversifolia* leave.

Treatments	Spleen index	Thymus index	Phagocytic index	Energy metabolism	Nitric oxide (mg/ml)	Oxygen radicals (mmol/ml)
Neg. control	0.0048 ± 0.0007^a^	0.00042 ± 0.00013	0.41 ± 0.58^a^	0.029 ± 0.003^a^	0.09 ± 0.006^a^	51.23 ± 13.32^a^
Normal control	0.0098 ± 0.0008^b^	0.00109 ± 0.00012	1.11 ± 0.34^b^	0.098 ± 0.008^b^	1.34 ± 0.0027^b^	297.40 ± 1.31^b^
A-L (5 mg/kg)	0.0191 ± 0.005^c^	0.00077 ± 0.00035	4.45 ± 1.18^c^	0.13 ± 0.01^c^	1.49 ± 0.034^b^	357.01 ± 8.167^b^
50 mg/kg extract	0.0085 ± 0.004^ab^	0.00078 ± 0.00045	2.15 ± 0.54^a^	0.26 ± 0.05^d^	0.33 ± 0.08^c^	110.34 ± 7.73^c^
100 mg/kg extract	0.0170 ± 0.002^c^	0.00078 ± 0.00046	2.93 ± 0.54^d^	0.12 ± 0.03^c^	0.52 ± 0.15^c^	123.13 ± 35.74^c^
ANOVA significance	*p*=0.0014; *F* = 9.549	*p*=0.62; *F* = 0.67	*p*=0.003; *F* = 13.734	*p*=0.0004; *F* = 12.966	*p* < 0.0001; *F* = 132.17	*p* < 0.0001; *F* = 118.96

Data are expressed as mean ± SD; *n* = 5. Subscript letters indicate the difference within the group following the Student's post hoc test with *p* < 0.05. A–L: atermether 80 mg/lumefantrine 480 mg (5 mg/kg); nor-control: non-immunosuppressed animals without any treatment; neg. control: immunosuppressed animals treated with distilled water only.

## Data Availability

Data are available from the corresponding author upon request.
